# The modification of the thrombin generation test for the clinical assessment of dabigatran etexilate efficiency

**DOI:** 10.1038/srep29242

**Published:** 2016-07-05

**Authors:** Irina V. Gribkova, Elena N. Lipets, Irina G. Rekhtina, Alex I. Bernakevich, Dorzho B. Ayusheev, Ruzanna A. Ovsepyan, Fazoil I. Ataullakhanov, Elena I. Sinauridze

**Affiliations:** 1 National Research Center for Hematology, Novyi Zykovskii pr., 4, Moscow, 125167, Russia; 2 Center for Theoretical Problems of Physicochemical Pharmacology, Russian Academy of Sciences, Kosygina street, 4, Moscow, 119991, Russia; 3 Central Institute of Traumatology and Orthopedics named after N.N. Priorov, Priorova Str., 10, Moscow, 127299, Russia; 4 Federal Research and Clinical Centre of Pediatric Hematology, Oncology and Immunology named after Dmitriy Rogachev, Ministry of Health, Samory Mashela Str., 1, Moscow, 117997, Russia; 5 Hematological Corporation “HemaCore” LLC, Naychnyi proezd 20, building 2, Moscow, 117246, Russia; 6 M.V. Lomonosov Moscow State University, 1 Lеninskie gory St., Moscow, 119991, Russia

## Abstract

A new oral anticoagulant, dabigatran etexilate (DE, a prodrug of direct thrombin inhibitor (DTI) dabigatran), has been used clinically to prevent thrombosis. The assessment of dabigatran efficiency is necessary in some clinical cases, such as renal insufficiency, risk of bleeding, and drug interactions. However, a specific thrombin generation test (TGT) that is one of the most informative and sensitive to anticoagulant therapy (calibrated automated thrombinography (САТ)) shows a paradoxical increase of test parameters, such as endogenous thrombin potential (ETP) and peak thrombin, in patients receiving DE. The paradoxical behaviour of ETP and peak thrombin in these patients in the presence of DTIs is mostly caused by a decrease in the activity of thrombin in the α_2_-macroglobulin-thrombin complex that is used as a calibrator in CAT. For a correct estimation of the TGT parameters in patient’s plasma containing DTIs we proposed to use our previously described alternative calibration method that is based on the measurement of the fluorescence signal of a well-known concentration of the reaction product (7-amino-4-methylcoumarin). In this study, the validity of such approach was demonstrated in an *ex vivo* study in patients with knee replacement and two special patients with multiple myeloma, who received DE for thrombosis prophylaxis.

Currently, many preparations (heparins, vitamin K antagonists (VKAs), new oral anticoagulants) are used clinically to prevent thrombosis. Dabigatran etexilate (DE), a prodrug of the low molecular weight direct thrombin inhibitor dabigatran^1^, has become increasingly popular^2^,^3^,^4^,^5^,^6^. It is a new type oral anticoagulant. Advantage of this preparation compared to the VKAs, which have been the only type of oral anticoagulants until recently, is that it rapidly and directly target thrombin once formed and in contrast to VKAs does not interfere in the synthesis of vitamin K dependent coagulation factors. In addition, dabigatran may show a lower number of food and drug interactions^7^,^8^. Moreover, dabigatran has a predictable pharmacokinetic profile^9^,^10^ and wide therapeutic-window, allowing for a fixed-dose regimen without the need for routine coagulation monitoring^8^. However it has recently become increasingly clear that there are some clinical situations (renal insufficiency, risk of bleeding, and drug interactions) in which clinicians will need to make an assessment of the anticoagulant status of a patient receiving DE^11^,^12^,^13^. It may even be vitally important^12^,^14^,^15^.

Many studies have been dedicated to the possibility of an assessment of dabigatran efficiency using different laboratory tests^11^,^14^,^16^,^17^,^18^,^19^,^20^. Not all the methods in use are appropriate for this task. Thrombin time (TT) can produce quantitative results only for low concentrations, as it is an over-sensitive test^15^,^18^,^19^,^21^,^22^. The activated partial thromboplastin time can only give an approximation of dabigatran activity at low concentrations but is not linear at dabigatran concentrations used clinically^18^,^19^,^21^,^23^. International normalized ratio (or prothrombin time) have low sensitivity to DTIs^18^,^19^,^21^,^23^.

Hemoclot Thrombin Inhibitor (HTI) (or dilute TT) may be a good test for the assessment of dabigatran efficiency. HTI showed excellent linear correlation at all doses^11^,^16^,^18^. But it is not a global test, i.e. it is suitable to measure dabigatran concentrations but it cannot show the status of the coagulation system as the whole. In particular, it does not show reversal of dabigatran action by prothrombin complex concentrate administration^24^. The global coagulation test of thrombin generation (TGT) developed and modified by H.C. Hemker (calibrated automated thrombinography, САТ)^25^ is a sensitive method for an assessment of the anticoagulant therapy, reflecting most if not all of coagulation system’s functions^26^,^27^,^28^,^29^. This test determines the kinetics of the appearance of active thrombin in plasma after the activation of coagulation. The basic TGT parameters are: endogenous thrombin potential (ETP)–the sum amount of active thrombin which was present in the system over entire time of experiment (area under the thrombin generation curve), maximal thrombin concentration in the sample (peak thrombin), time to reach this maximal concentration (tmax), and lag-time before the beginning of accelerated thrombin production (t-lag), which is by convention determined as the time before the thrombin concentration in the sample reaches 10 nM. In the presence of anticoagulants, the first two parameters decrease, while tmax and t-lag increase.

In spite of the high sensitivity of TGT to the presence of different anticoagulants, the capabilities of this test in the assessment of DE application *in vivo* are not evident^8^. The analysis of DE application for the prophylaxis of thrombosis in patients after hip or knee replacement in one study^30^ showed a paradoxical increase of ETP and peak thrombin (i.e. an apparent enhancement of thrombin generation) and simultaneously the expected change (prolongation) of two other test parameters tmax and t-lag (i.e. inhibition of initiation of thrombin generation). Similar results were recently obtained in another study in patients following orthopedic surgery and in patients with atrial fibrillation^31^. Thus, the application of TGT in the presence of some anticoagulants is complicated and requires additional study^8^.

The reasons for the irregular behavior of some TGT parameters in CAT in the presence of dabigatran are not well defined. In course of coagulation along with other reactions thrombin binds with α_2_-macroglobulin. Thrombin in this complex loses its capacity to interact with fibrinogen, but not with the low molecular weight substrates. In this complex it cannot be subjected to high molecular weight anticoagulants like heparins, making CAT an excellent method for monitoring them. However, dabigatran is a low molecular weight compound, and it can interact with free thrombin as well as with thrombin in the α_2_-macroglobulin-thrombin complex (α_2_MT). Wagenvoord *et al*.^32^ suggested that spuriously increased values of ETP and peak thrombin could be a consequence of the incorrect algorithm (for dabigatran samples) that is used in CAT to subtract α_2_MT activity from the total amidolytic activity in the experimental wells. On the other hand, dabigatran will also inhibit α_2_MT that is used as calibrator reagent in CAT and that is added to calibrator wells containing patient plasma as well. Dabigatran in the calibrator wells will decrease the value of the calibration signal and as a consequence thrombin values are calculated that are erroneously too high. As an alternative calibration approach that allows the measurement of TGT in patients receiving DE, we propose the use of a solution of 7-amino-4-methylcoumarin (AMC) in a well-defined concentration as a calibration standard. AMC is the fluorescent product of the hydrolysis reaction of the fluorogenic substrate used in CAT. Recently we successfully used the proposed method *in vitro* to examine a number of new DTIs^33^. Aim of the present study is to explore whether this modified calibration protocol is applicable for the measurement of thrombin generation in samples from dabigatran-treated individuals.

## Results

### Effect of DTIs on the activity of the thrombin calibrator used in CAT

**Figure 1:**
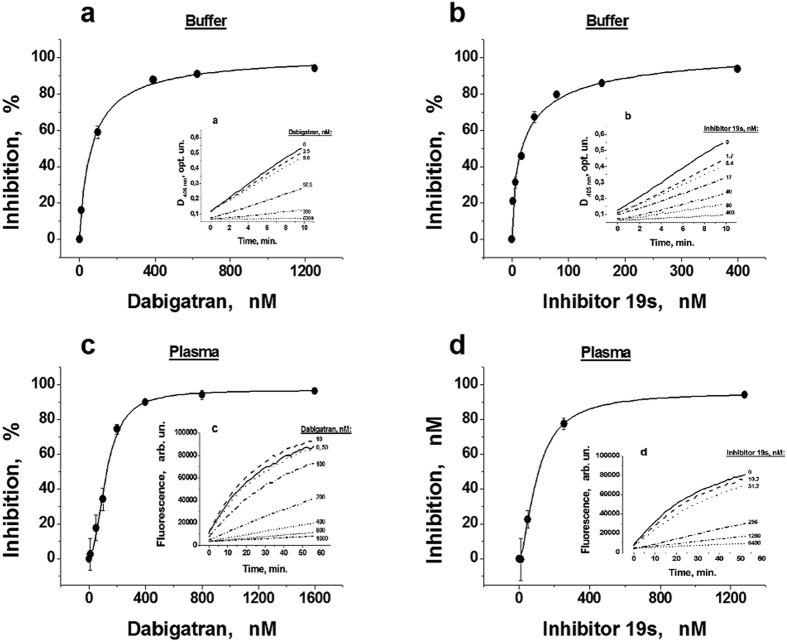
Effects of DTIs on thrombin calibrator (ThrCal) activity in a buffer system and plasma. (**a**,**b**) The percentage of inhibition for the hydrolysis rate of thrombin-specific chromogenic substrate by ThrCal (0.73 nM) in buffer solution in the presence of different concentrations of dabigatran or 19s, respectively. (**c,d**) The percentage of inhibition for the hydrolysis rate of thrombin-specific fluorogenic substrate in donor plasma in the presence of a constant ThrCal concentration (116.8 nM) and different concentrations of dabigatran or 19 s, respectively. Different plasma sets were used in the groups of different inhibitors. The inserts on the panels show the typical examples of product’s accumulation for corresponding experiments.

To determine the effect of DTIs on the activity of ThrCal used in CAT the rates of thrombin-specific substrate hydrolysis in the presence of a constant ThrCal concentration and different concentrations of two DTIs (dabigatran or 19 s) were measured both in buffer solution and plasma. Chromogenic substrate was used in buffer, whereas the fluorogenic substrate, commonly applied in CAT, was used in plasma. Different sets of plasma samples were used in each type of experiments. The results are shown in Fig. 1.

It is clear that in buffer solution, both dabigatran (Fig. 1a) and 19 s (Fig. 1b) inhibit the rate of chromogenic substrate hydrolysis by ThrCal in a concentration-dependent manner. The averaged values of the inhibition of the substrate hydrolysis rate (in %) for different concentrations of thrombin inhibitors are presented (mean ± SD, n = 5). Similarly, dabigatran and 19 s inhibit the accumulation of fluorescent product of thrombin-specific fluorogenic substrate hydrolysis by ThrCal in plasma. Dependencies of inhibition of AMC accumulation on the concentrations of DTIs in plasma are presented in Fig. 1c,d.

### Effect of DTIs on the fluorescence of AMC

To eliminate the influence of DTIs on ThrCal activity (used in CAT for calibration), the calibration of fluorescent signals was performed by measuring the fluorescence of a well-defined concentration of hydrolysis product (AMC). The fluorescence of a constant concentration of AMC (8 μM) in the presence of different concentrations of these inhibitors was measured in the plasma of 5 donors (different for each inhibitor), and the fluorescence values for 1 μM of AMC were calculated. All the measurements were carried out in the presence of a constant concentration of fluorogenic substrate (400 μM) to emulate the conditions of TGT. The value of 1 μM AMC fluorescence (for each plasma sample) did not change in the presence of DTIs. It amounted to 723 ± 14, and 719 ± 26 arbitrary units (arb. un.) for dabigatran and 19 s, respectively, in the range of concentrations of dabigatran up to 1.6 μM, or 19 s up to 6.4 μM. No significant difference was observed between these values for both inhibitors (ANOVA, *P* > *0.05*). This value was defined as the calibration value for the calculation of real AMC concentrations in plasma for each time point.

### Parameters of TGT (method CAT) in the presence of DTIs

**Figure 2:**
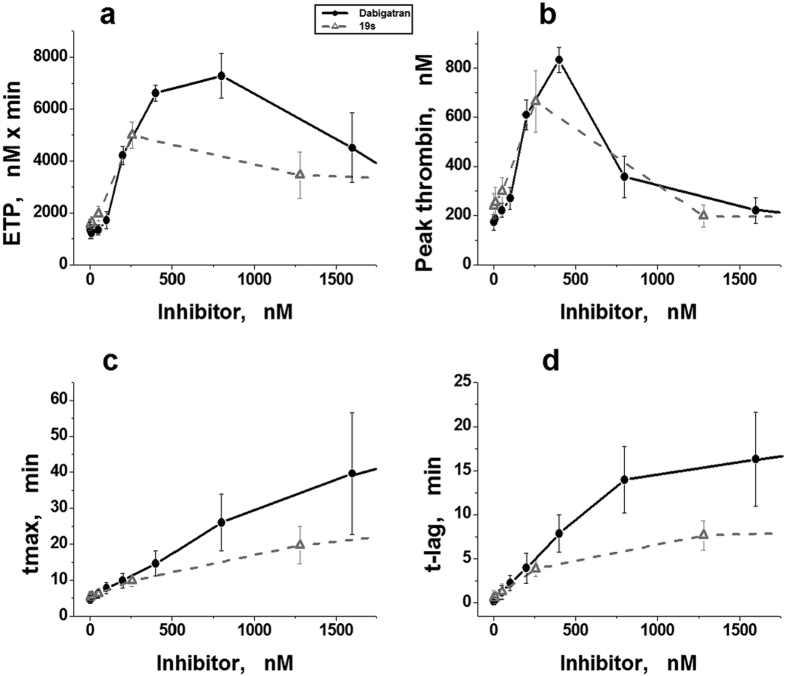
Parameters of TGT in the presence of DTIs determined by CAT in donor plasma. (**a–d**) Endogenous thrombin potential (ETP), maximal thrombin concentration in the sample (peak thrombin), time to maximal concentration (tmax), and time to thrombin concentration 10 nM (t-lag) plotted against concentrations of dabigatran (•, black solid line), or thrombin inhibitor 19 s (Δ, grey dashed line). Mean values ± SD (n = 5) are presented. Different plasma sets were used in the groups of different inhibitors.

TGT in the presence of different concentrations of two DTIs (dabigatran or 19 s) was performed by CAT using the plasma of 5 donors (different for the each inhibitor group). The results obtained (mean ± SD, n = 5) are shown in Fig. 2. One can see that at concentrations of up to ~400 nM for dabigatran and ~256 nM for 19 s, values of ETP and peak thrombin increase significantly. These parameters begin to decrease with further increase of both inhibitors concentrations (Fig. 2a,b). At the same time the parameters tmax and t-lag increase droningly with increasing DTI concentrations at all investigated concentrations (Fig. 2c,d).

### Parameters of TGT in the presence of DTIs measured upon calibration of the signals using AMC fluorescence

**Figure 3:**
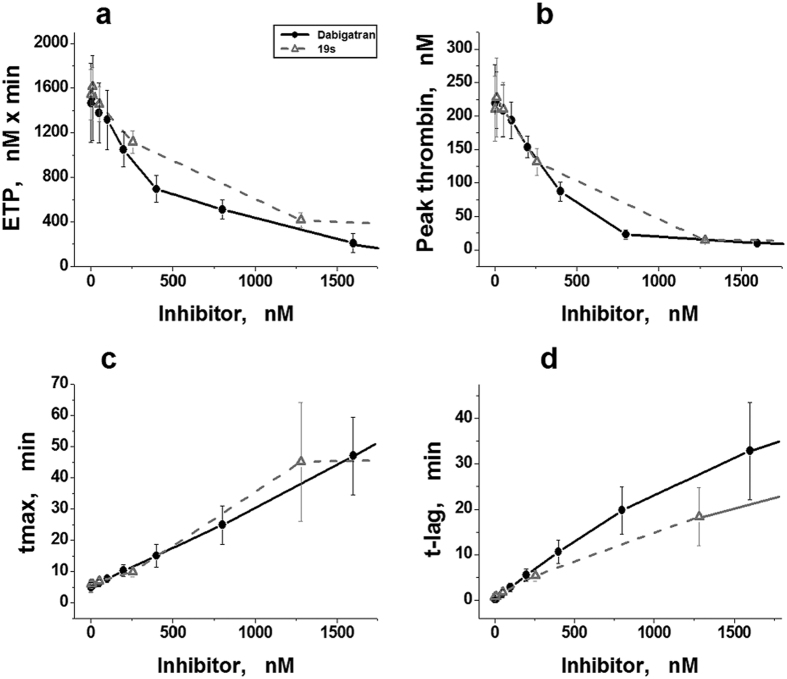
Parameters of TGT in donor plasma in the presence of DTIs, determined by a modified method using the calibration of signals by AMC fluorescence. (**a–d**) Endogenous thrombin potential (ETP), maximal thrombin concentration in the sample (peak thrombin), time to maximal concentration (tmax), and time to thrombin concentration 10 nM (t-lag) plotted against concentrations of dabigatran (•, black solid line) or the thrombin inhibitor 19s (Δ, grey dashed line). Mean values ± SD (n = 5) are presented. Different plasma sets were used in the groups of different inhibitors, but they were the same as in the groups presented in Fig. 2.

In parallel to CAT, parameters of TGT were determined from the same donor plasma samples in the presence of dabigatran or 19 s by the modified method with calibration of the fluorescence signal by AMC fluorescence in each plasma sample. The results obtained are presented in Fig. 3.

Like the results obtained with CAT, the values of tmax and t-lag increased dose-dependently with increasing concentrations of thrombin inhibitor (Fig. 3c,d). At the same time, the behaviour of ETP and peak thrombin differed from the results obtained with CAT.

After signal calibration by AMC fluorescence, a very small increase (no more than 2–5% of an initial value) was observed in these parameters at low concentrations of both inhibitors (~10 nM). This increase, however, was insignificant (ANOVA, *P* > 0.05). The values of ETP and peak thrombin decreased dose-dependently with further increase in dabigatran or thrombin inhibitor 19s concentration (Fig. 3a,b).

### Comparison of two modifications of TGT in donors’ plasma

**Figure 4:**
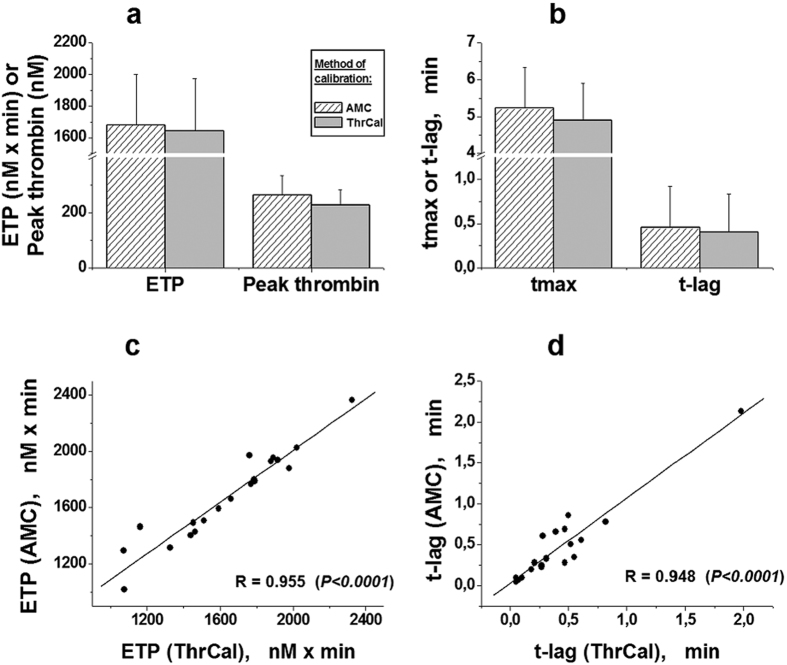
Comparison of the TGT parameters obtained using two different method of fluorescent signal calibration in 20 plasma samples in the absence of DTIs. The values of mean ± SD (n = 20) for ETP and peak thrombin (**a**), as well as for tmax and t-lag (**b**) are presented. The examples of the coefficients of linear regression for results obtained using different methods of calibration (by ThrCal, or AMC) for ETP (**c**), and t-lag (**d**). No significant differences were observed (ANOVA, P > 0.05, or paired t-test of Student, P > 0.05) for these parameters.

To compare CAT and the proposed modified method, we carried out measuring of thrombin generation in parallel by both of these methods in plasma of 20 donors in the absence of DTI (Fig. 4). The normality of parameters’ distribution has been examined beforehand using the D’Agostino-Pearson test in program MedCalc Statistical Software bvba (version 14.12) (USA, Belgium). All of the parameters in both groups were normal distributed. One-way analysis of variance (ANOVA) followed by Tukey’s multiple comparison test was used for statistical analysis to compare two groups, with a P-value < 0.05 considered to be statistically significant. There were no significant differences for all of the parameters (Fig. 4a,b). The linear regressions were constructed for each parameter measured by both, CAT and our modified TGT method. The coefficients of correlation of these linear regressions were high enough. As the examples, the linear regressions for ETP (Fig. 4c) and t-lag (Fig. 4d) are presented.

### Comparison of two modifications of TGT in patients receiving DE

A comparison of the TGT parameters obtained in parallel by both CAT and the proposed modified method was carried out in two patients with a recurrence of multiple myeloma and severe renal failure, who received chemotherapy (patients 1 and 2), and seven patients with knee replacement (patients 3–9). All these patients received DE for the prophylaxis of thrombosis.

#### Patient 1

**Figure 5:**
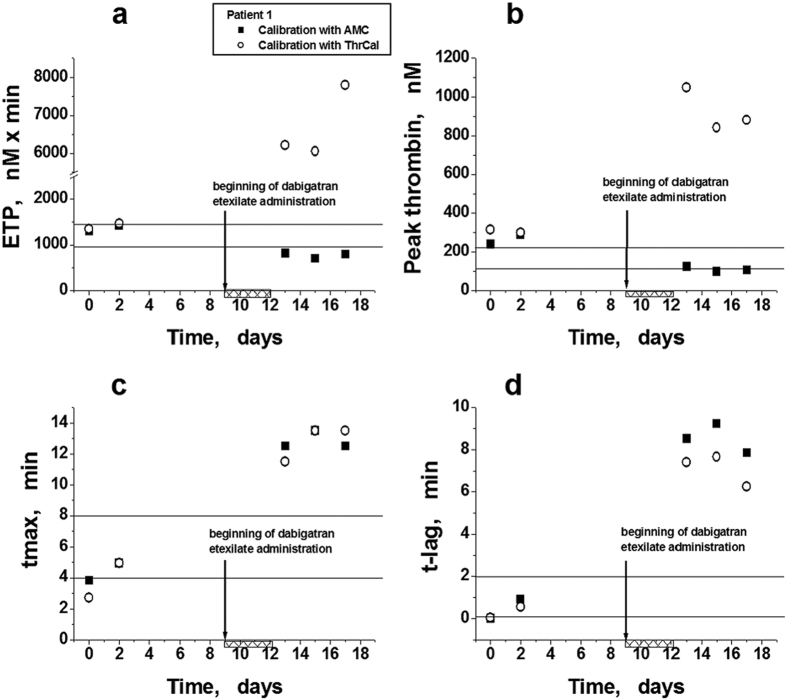
Parameters of TGT determined *ex vivo* in patient 1 using two variations of the method. (**a**–**d**) Endogenous thrombin potential (ETP), maximal thrombin concentration in the sample (peak thrombin), time to maximal concentration (tmax), and time to thrombin concentration 10 nM (t-lag) plotted against time. TGT was measured by CAT (○) and by the modified method with a signal calibration by AMC fluorescence (■). The beginnings of chemotherapy and dabigatran etexilate administration are denoted by arrows. Shaded rectangles show the days of chemotherapy. Horizontal lines note the areas of the previously determined reference values for each parameter (mean ± SD, n = 50).

Along with chemotherapy (thalidomide 100 mg/day and dexamethasone 40 mg/day), patient received prophylactic dose of DE (110 mg) once per day. Thrombin generation was examined after one course of chemotherapy. The modified method showed that this dose of preparation stably supported ETP in the decreased state compared with normal values determined previously in donors’ plasma (n = 50) (Fig. 5a), and it decreased the initially heightened values of peak thrombin to the lower limit of the reference values (Fig. 5b). Both CAT and the modified method showed similar values for ETP and peak thrombin in the absence of DE (the first and second points in Fig. 5a,b), but these parameters increased in CAT after the administration of the preparation (Fig. 5a,b) because of inhibition of the calibrator (ThrCal) activity. Both methods showed approximately equal prolongation of tmax and t-lag in the presence of DE (Fig. 5c,d).

#### Patient 2

The state of haemostasis of patient 2 was followed over a long time period. For this time period, patient received 2 courses of chemotherapy, including thalidomide (100 mg/day) and dexamethasone (40 mg/day), for 4 days with 28-day intervals between courses. In the following course, thalidomide was replaced by lenalidomide (5 mg/day). Anticoagulant therapy proceeded during all periods of supervision. Patient received prophylactic dose of DE (110 mg) once per day.

**Figure 6:**
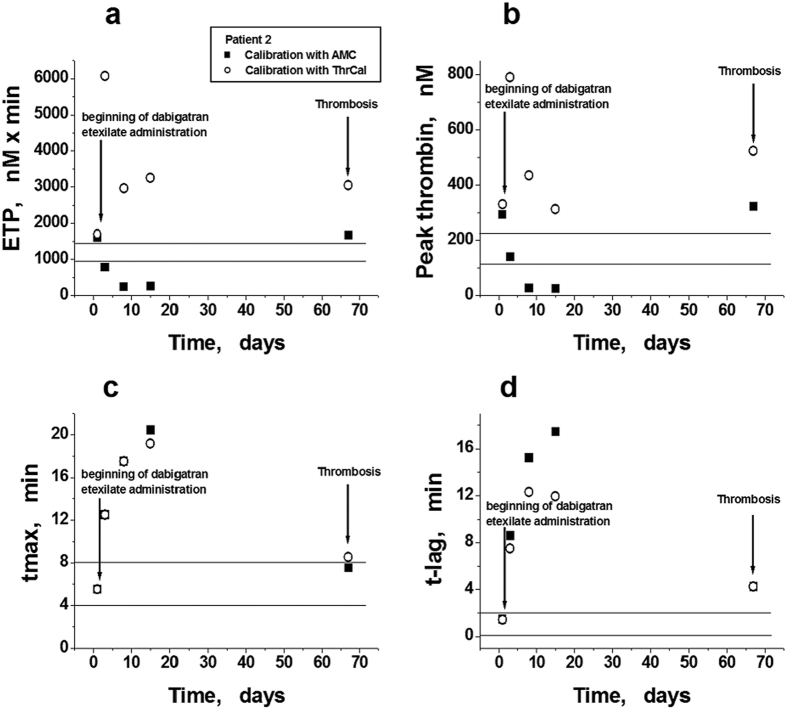
Parameters of TGT determined *ex vivo* in patient 2 using two variations of the method. (**a**–**d**) Endogenous thrombin potential (ETP), maximal thrombin concentration in the sample (peak thrombin), time to maximal concentration (tmax), and time to thrombin concentration 10 nM (t-lag) plotted against time. TGT was measured by CAT (○) and by the modified method with a signal calibration by AMC fluorescence (■). The beginning of dabigatran etexilate administration and moment of thrombosis detection are denoted by arrows. Horizontal lines note the areas of the previously determined reference values for each parameter (mean ± SD, n = 50).

The thrombosis of arteriovenous fistula was observed one day after changing the chemotherapy regimen. The results of the parallel measurements of TGT performed by two different methods are presented in Fig. 6.

The first points in all the panels of the figure correspond to the initial values of each parameter before the beginning of chemo- and anticoagulant therapy. After the first course of chemotherapy and the start of DE administration, the modified TGT showed a decrease in the heightened initial values of ETP and peak thrombin to values significantly lower than normal values, and maintained them at these levels (Fig. 6a,b). In the third course of chemotherapy, after switching to lenalidomide, when the patient obtained thrombosis, the values of ETP and peak thrombin determined by the modified method were increased relative to reference values (Fig. 6a,b).

CAT showed increased values of ETP and peak thrombin at the initial time point (without DE), which were similar to the corresponding values determined by the new variant of TGT. However, after DE, the values of these parameters did not decrease; in fact, they increased (Fig. 6a,b) and were maintained at levels higher than normal values during the entire therapeutic period. The appearance of thrombosis did not change ETP and only slightly increased peak thrombin (Fig. 6a,b).

The parameters tmax and t-lag changed equally upon TGT by both methods. They were prolonged after DE administration, but shortened (up to normal or slightly increased values) upon thrombosis detection (Fig. 6c,d).

#### Patients 3–9

**Figure 7:**
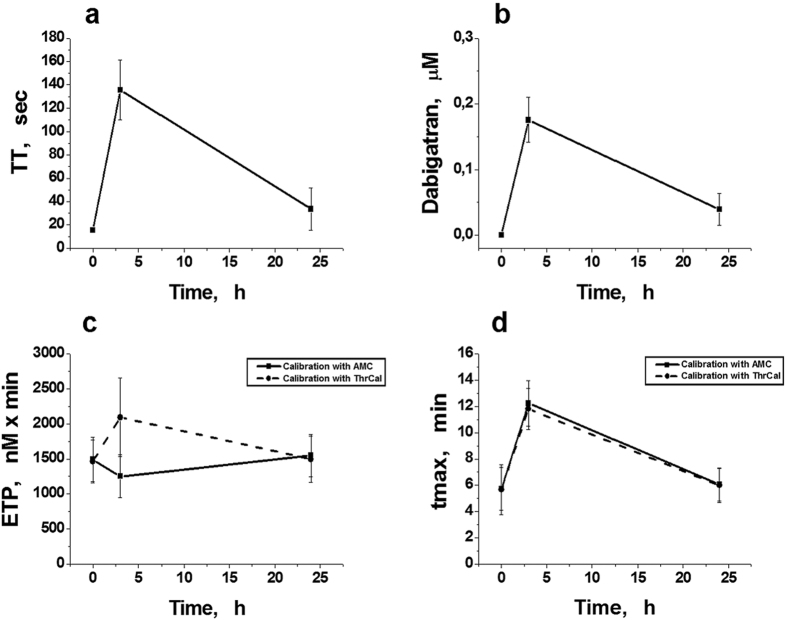
Parameters of TGT determined *ex vivo*, using two variations of the method, in 7 patients, who received DE after knee replacement surgery. The TGT was measured before DE, as well as 3 and 24 h after the first administration of 220 mg DE. (**a**) Thrombin times of clotting in different time points. (**b**) The concentrations of dabigatran in plasma of each patient, calculated accordingly the study^9^. (**c**,**d**) Endogenous thrombin potential (ETP) and time to maximal concentration (tmax), respectively, plotted against time. TGT was measured by CAT (dash lines) and by the modified method with a signal calibration by AMC fluorescence (solid lines). Mean values ± SD (n = 7) are presented.

For these patients with knee replacement surgery TT was measured in all of the investigated points (before, as well as 3 and 24 h after surgery) (Fig. 7a). We were able to roughly estimate the concentration of dabigatran in plasma, using the results of the study^9^ (Fig. 7b). The panels c, d in Fig. 7 show the parameters of the TGT, obtained using our modified method with calibration by AMC (the solid lines), and with the regular CAT method with calibration by ThrCal (the dash lines) in the patients who received DE (220 mg) in the first time (the next morning after operation). ETP (Fig. 7c) and peak thrombin (data not shown) decreased 3 hours after administration of DE if they were determined by the modified method, and then increased again (24 h after the first administration). An increase in dabigatran concentration corresponded to decrease in these parameters (Fig. 7b,c). At the same time, these parameters measured by CAT increased 3 hours after receiving DE, and then decreased again after 24 h (Fig. 7c). The parameters tmax (Fig. 7d) and t-lag (data not shown) changed in parallel for both modifications of the TGT as it was expected.

## Discussion

The possibility of inhibition of α_2_MT complex (ThrCal) in the presence of DTIs has been shown in ref. 32 and has been assumed in recent works, where the assessment of the efficiency of the clinical application of DE using TGT has been discussed^21^,^24^. However in contrast to plasma of patients receiving DE, plasma in the study^32^ has not contained DTI (AR-H067637) in the calibration wells, and the authors have observed only a low ETP increase (~10–15%) at low DTI concentrations (<100 nM). That has been explained by incorrect algorithm for calculation of α_2_MT level in the experimental wells in the presence of a reversible competitive inhibitor. The authors even have concluded that the artificial increase in ETP at low inhibitor concentrations disappears, if the first part of an area under thrombin generation curve (until maximum thrombin concentration in the sample) is used for characterization of the thrombin generation, and therefore, this method may be well suited for a characterization of the thrombin generation in the plasma of patients. However, it is not so. The authors of 32 have not considered the question, that the presence of DTI in the starting plasma of patients should reduce an activity of ThrCal, which has been introduced into this plasma in the calibration wells, and thus should lead to a complete inability to use CAT for the patients’ plasma with DTIs. The authors of 32 could not see this effect, because to measure the calibration signal they used the plasma without added thrombin inhibitor. We assumed and demonstrated experimentally that the main reason for the high paradoxical increase of ETP and peak thrombin in CAT in patients receiving DTIs was an inhibition of ThrCal in the calibration wells. It was shown that DTIs decrease the activity of ThrCal both in buffer solution (Fig. 1a,b) and in plasma (Fig. 1c,d). The use of this complex as a thrombin-calibrator in the presence of DTIs results in a decrease of the value of calibration signal (counting on the thrombin activity which is given in an instruction to the used reagent), and therefore in the overvaluation of the parameters ETP and peak thrombin in the presence of the same total signal values (Fig. 2a,b). At the same time, the time parameters of TGT (tmax and t-lag) were prolonged with increased concentrations of inhibitors (i.e. they were changed in the expected direction) (Fig. 2c,d). This was observed in a study^30^ that measured TGT by CAT in patients receiving DE (220 mg/day) for thromboprophylaxis. On the second day after total hip/knee replacement surgery, ETP and peak thrombin in these patients increased approximately two times; at the same time, tmax and t-lag were significantly prolonged. To explain this fact, the authors refer to the work^32^. However, it is clear that the effect obtained in^30^ cannot be explained completely by these reasons. This effect is much greater (ETP increases twice) and it is reached at much higher concentrations of inhibitor (corresponding to^34^, dabigatran concentration in patient plasma after DE administration (150 mg bid or 220 mg once daily can reach up to 184 μg/l (390 nM), but in some cases, this concentration may reach up to 408 μg/l (865 nМ)^17^). The effects observed in our study conform to those observed in^30^: ETP increases two-fold at a dabigatran concentration of 140 nM and continues to increase with further increases in concentration up to ~800 nM (Fig. 2a).

Similar measurements of the CAT parameters in *in vitro* experiments were performed in refs 11,24, but in these studies, no increase of ETP and peak thrombin in the presence of dabigatran was observed. This may be because in these experiments, dabigatran also was not added to the calibration wells. Thus, the effect of ETP increase observed in the work^32^ was different from the effect we observed. These effects could be explained by different reasons. It is clear that signal calibration without the presence of thrombin inhibitor in calibration wells may be used *in vitro* when different concentrations of thrombin inhibitors (including dabigatran) are added to the plasma during the experiment. However, such an arrangement should be excluded upon the measurement of thrombin generation in patients whose plasma already contains this inhibitor in unknown concentrations. Thus, the proper measurement of the calibration signal for the plasma of each patient is necessary, since the calibration fluorescence signal in САТ depends on both the colour and composition of plasma and on the concentration of present thrombin inhibitor (dabigatran).

To adapt TGT for the measurement of the influence of DTIs on the coagulation system, we proposed another method for the calibration of a fluorescent signal to calculate the actual AMC concentration in plasma at each time point. The well-determined concentration of AMC (8 μM) was used as standard for these measurements, and the fluorescent signal of 1 μM of AMC was calculated for each specific plasma. In separate experiments, it was shown that the size of this signal does not depend on the concentration of inhibitor in plasma (see Results).

We carried out *in vitro* comparative research on the TGT parameters in the same plasma samples, in the absence (n = 20), or in the presence (n = 10) of various concentrations of added thrombin inhibitors (dabigatran and 19s) using both methods of calibration. The results showed that all TGT parameters obtained in the same plasma samples with different methods of calibration in the absence of thrombin inhibitors were practically identical (Fig. 4). The coefficients of a linear regression were >0.88 (*P* < 0.0001). At the same time, unlike САТ, when calibrating a signal using the value of AMC fluorescence, ЕТР and peak thrombin decreased with the increase of thrombin inhibitor concentration in plasma, as expected (Figs 2 and 3). We also observed a very low increase in ЕТР at low (less than 100 nM) concentrations of inhibitor. This effect was less than 10% and could be explained by the use an improper algorithm for the subtraction of the amidolytic activity of α_2_MT complex from the full amidolytic activity of the sample, according to^32^.

To check the efficiency of our modification of signal calibration in TGT in the presence of DTIs, plasma samples of two patients with multiple myeloma receiving chemotherapy were investigated from the beginning of anticoagulant therapy with DE used for thromboprophylaxis. TGT was performed in parallel using two methods: САТ (calibration by means of ThrCal) and the modified method (calibration using the fluorescence of a known AMC concentration) (Figs 5 and 6).

After changing the chemotherapy regimen, the thrombosis was fixed in patient 2 (with a background of the same dose of DE) (Fig. 6). CAT demonstrated increasing ETP and peak thrombin in both patients during DE administration. ETP was practically unchanged and peak thrombin increased when thrombosis occurred (Fig. 6). At the same time, the modified method fixed the decrease of ETP and peak thrombin in the presence of DE, and these parameters immediately increased in patient 2 upon thrombosis (Figs 5 and 6). The parameters tmax and t-lag behaved equally with both methods of calibration (Figs 5 and 6) and were not essentially shorter in comparison to normal values in the moment of originated thrombosis (Fig. 6). This indicates that the estimation of the action of DTIs by the TGT parameters tmax and t-lag may be ineffective. This situation can be explained by the fact that sometimes it is difficult to accurately measure tmax and t-lag and that larger variations exist in these parameters in the whole population (СV_total_ ~ 30%) than for ETP and peak thrombin (CV_total_ ~ 15%)^35^,^36^. The parameters ETP and peak thrombin could be more convenient for analysis.

All these conclusions concerning different methods of fluorescent signal calibration were also confirmed in seven other patients, who received DE after total knee replacement surgery (Fig. 7). Peak thrombin and ETP in these patients, which were obtained, using the modified TGT method, showed the correlation with thrombin time (and with dabigatran plasma concentration which was roughly estimated using TT).

Thus, our investigation confirms the inhibitory action of DTIs on the activity of ThrCal, as well as the possibility of applying a new method for signal calibration in TGT to measure the efficiency of thrombin generation in plasma of patients receiving DE. However, our study presents limitations that need to be acknowledged. We comprehensively showed the efficacy of our callibration method in an *in vitro* study, but only 9 patients actually treated with DE were presented. In addition, two of the most informative cases were patients with multiple myeloma and terminal renal failure receiving dialysis, which is known to affect DE levels. We acknowledge that treatment with DE is not recommended in such patients (see, for example ref. 37), but enrollment of these patients in this investigation was approved due to very specific clinical conditions which associated a high risk venous thromboembolism and transient contraindications to heparin or VKAs. We understand that severe renal failure and dialysis can significantly change dabigatran levels, and we were not able to measure these concentrations in this study. However, since we compared both methods in parallell using the same samples, we believe that the influence of this variation is not critical to our conclusions. Thus, our results suggest that this new callibration method is promising, but more extensive testing in different clinical contexts is warranted before it can be potentially incorporated into clinical practice in the future.

## Materials and Methods

### Ethics Statement

This study was approved by the Ethical Committee of the Center for Theoretical Problems of Physicochemical Pharmacology (Permit Number: 21-04-2009)). All methods were carried out in accordance with the approved guidelines. All participants provided written informed consent before blood collection.

### Donors and patients

Donors’ blood was collected at the Blood Transfusion Station of the National Research Center for Hematology and was used without identification in *in vitro* experiments.

Nine patients, who received DE for thrombosis prophylaxis, were included in the *ex vivo* investigation.

Two patients (number 1 and 2) with the recurrence of multiple myeloma and the terminal stage of renal failure received courses of chemotherapy by program: thalidomide (100 mg per day) and dexamethasone (40 mg per day). Patient 1 received one course of chemotherapy. In patient 2, thalidomide was replaced by lenalidomide (5 mg per day) after two courses. Because of high rates of renal failure, both patients were subjected to haemodialysis. Unfractionated heparin (5000–7500 IU) was administered during haemodialysis. For the prophylaxis of thrombosis, patients received dabigatran etexilate (110 mg per day) during the time of supervision. DE is not recommended in the case of severe renal failure. However an anticoagulant prophylaxis was needed since these patients received chemotherapy with thalidomide or lenalidomide combined with dexametasone. But warfarin and heparins could not be used in these specific patients, therefore it was necessary to test the other anticoagulants. Blood for haemostasis analysis by TGT (in 2 modifications) was collected before haemodialysis into vacuum tubes (Vacuette) with 3.8% (0.129 M) sodium citrate at a blood/anticoagulant ratio of 9/1. Platelet poor plasma (PPP) for tests was prepared by centrifuging blood for 15 min at 1600 g. Clinically apparent thrombosis of arteriovenous fistula was recorded one day after changing the course of chemotherapy in patient 2.

Seven other patients (numbers 3–9) were after knee replacement surgery and received DE in accordance with the currently used guidelines .These patients were investigated before DE, as well as 3 and 24 h after the first dose of DE (220 mg), which was obtained 24 h after knee replacement surgery. The platelet free plasma (PFP) was used for TGT, obtained by double centrifugation (1600 g × 15 min, and then 10000 g × 5 min). Thrombin time in the samples of these patients was measured using Stago STA Compact Coagulation Analyzer (Diagnostica Stago, USA) with reagents kit of Renam (Moscow, Russia).

### Reagents

The thrombin-specific chromogenic substrate Tosyl-Gly-Pro-Arg-p-nitroanilide (Chromozym-TH) was purchased from Sigma (USA).

For the thrombin generation test, the following reagents were used: thrombin-specific fluorogenic substrate Z-Gly-Gly-Arg-AMC (Bachem, Switzerland); 7-amino-4-methylcoumarin, СаСl_2_, dimethyl sulfoxide (DMSO), and NaCl (Sigma-Aldrich, USA); thromboplastin (Renam, Russia); phosphatidylserine (from pig brain) and phosphatidylcholine (from egg yolk) produced by Avanti Polar Lipids Inc. (USA); and 4-(2-hydroxyethyl)-1-piperazine-2-ethanosulphonic acid (HEPES, Fisher Biotech, USA).

The calibrator for the determination of thrombin activity in the CAT test (ThrCal) was purchased from Thrombinoscope BV (the Netherlands) through Laborama (Russia).

Dabigatran (BIBR 953) was obtained from Selleckhem.com. HC-IOC-019s (C_6_H_5_-SO_2_-O-C_6_H_4_(CH_3_)-O-(CH_2_)_2_-NC_5_H_4_(NH_2_)) was synthesized at the Institute of Organic Chemistry of Russian Academy of Sciences (Russia)^33^. Pradaxa (dabigatran etexilate) (capsules 110 mg) was from Boehringer Ingelheim (Germany).

### Influence of DTIs (dabigatran and 19 s) on ThrCal activity in a buffer system

The activity of ThrCal (measured as units of thrombin activity) was determined from the rate of the hydrolysis of Chromozym-TH by ThrCal in the presence of different concentrations of DTI (dabigatran or 19 s).

Plate wells were filled with 171 μl of buffer A (20 mM HEPES (pH 8.0), 140 mМ NaCl), containing 0.1% polyethylene glycol (molecular weight 6000 Da). Thereafter, 4 μl of substrate was added to each well (final concentration 100 μM), followed by 5 μl of the solution of the thrombin inhibitor in DMSO (to a final concentration that was varied) and 20 μl of ThrCal (final concentration 0.73 nM). The hydrolysis rate was monitored spectrophotometrically at 405 nm (absorption maximum of the reaction product–*p*-nitroaniline) using Appliskan Multimode Microplate Reader (Thermo Scientific, Finland).

The initial hydrolysis rate was determined as the slope for the linear portion of the kinetic curve over the first 10–20 min of measurement. The reaction rate in the absence of inhibitor but with addition of the corresponding concentration of DMSO was taken as 100%. The inhibitory effect was expressed as the percentage reduction of the initial hydrolysis rate. Each result is the mean of two parallel determinations.

### Thrombin generation assay

The kinetics of thrombin generation in plasma was monitored using the hydrolysis rate of the slow fluorogenic substrate Z-Gly-Gly-Arg-AMC by thrombin being formed during clotting. This technique is based on the work of H.C. Hemker *et al*.^25^. Two modifications of this method were used, which differed only in the method of fluorescent signal calibration: by using ThrCal (CAT) or by signal of fluorescence of a well-known AMC concentration. The following parameters of the TGT were measured in each case: ETP, peak thrombin, tmax and t-lag.

Measurements were taken as follows. PPP was placed in the wells of a 96-well flat-bottom microtiter plate (80 μl/well). Thereafter, 20 μl of the fluorogenic substrate solution was added into each well, along with 5 μl of the thrombin inhibitor solution to be tested in different concentrations. In the wells without inhibitor, 5 μl of the solvent (DMSO) was added. The samples were incubated at 37 °C for 3–5 min, and thrombin generation was triggered with 20 μl of activator. Thromboplastin solution in buffer A, containing an additional 100 mM of СаСl_2_ and 25 μM of phospholipid suspension (20% of phosphatidylserine: 80% of phosphatidylcholine) was used as an activator of coagulation. Final concentrations of reagents in each sample were as follows: substrate, 400 μM; tissue factor, 5 pM; supplemented CaCl_2_, 16 mM; and phospholipids, 4 μM. Activator was added to all the wells simultaneously. The moment of activator addition was taken as time zero. The kinetics of the accumulation of the fluorescent reaction product (AMC) was continuously registered at 37 °С using an Appliskan Multimode Microplate Reader (Thermo Scientific, Finland) at λ_excitation_ = 355 nm and λ_emission_ = 460 nm. All the results are the averaged values of two parallel measurements.

The rate of product accumulation is proportional to the instant thrombin concentration in plasma. Therefore, the experimental curves of AMC accumulation were numerically differentiated in order to evaluate the thrombin generation curves. Data analysis was carried out using Origin 6.0 or 8.1 software (Microcal Software, USA).

The fluorescent signal calibration was performed by two different methods:

#### Calibrated automated thrombogram (CAT)

The thrombin generation assay by CAT was performed strictly as described previously by Hemker *et al*.^25^. The signal was calibrated by special thrombin calibrator, which represents α_2_MT complex (ThrCal). Separate calibration wells were used to determine the concentrations of active thrombin in the sample at each time point. They contained 80 μl of investigated plasma, 20 μl of fluorogenic substrate solution (final concentration 400 μM), additional CaCl_2_ (final concentration 16 mM), 20 μl of ThrCal with a final activity corresponding to the activity of 116.8 nM thrombin (according to specifications), and 5 μl of thrombin inhibitor solution in DMSO (in corresponding concentrations). Fluorescence signals in the experimental wells were normalized by the signal in the calibration well, thus allowing the calculation of thrombin concentration in the sample at each time point (taking into account an “inner filter” effect caused by changing AMC concentrations in the sample, and changes in the hydrolysis rate caused by substrate consumption during the reaction).

#### A new modification of the thrombin generation test

For the calibration of the fluorescence signal, two types of wells with non-clotting plasma were prepared. The first type contained 20 μl of buffer A instead of the activator to obtain the background plasma fluorescence level. The second (calibration wells) contained 10 μl of AMC solution in DMSO and 10 μl of buffer A instead of the activator. The final AMC concentration in these wells was 8 μM.

The background fluorescence was subtracted from the fluorescence signal in all of the wells. The value of fluorescence corresponding to 1 μM of AMC was recognized as the calibration value.

For calculation of a thrombin generation curve it is necessary to take into account:

- Nonlinearity of fluorescence intensity dependence on AMC concentration due to “inner filter” effect;
- Consumption of the substrate in course of the reaction;
- Contribution of amidolytic activity of α_2_MT in the total AMC concentration and, respectively, in measured fluorescence.

A more detailed description of the main steps of calculating the thrombin generation curve is presented below.

#### Determination of the dependence of AMC fluorescence on AMC concentration in the plasma (calculation of the “inner filter” effect)

A calibration curve was constructed by measurement of the AMC fluorescence in plasma in the presence of different AMC concentrations. We consider, that the concentrations of AMC and a substrate are equal to 0, and 400 μM, respectively, when the thrombin generation in the experimental wells begins, but then the substrate concentration gradually decreases and AMC, respectively, increases. Therefore, our calibration was performed in conditions of a constant sum of concentrations of AMC and the substrate (400 μM) in each point of the curve. The total volume of each sample was equal to 120 μl (80 μl plasma +20 μl of the substrate solution (of different concentrations) +10 μl of AMC solution in DMSO (in different concentrations) +10 μl of buffer A). The obtained curve was approximated by a function (1):


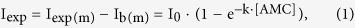


where I_exp(m)_ is a total measured fluorescence at certain [AMC] concentration; I_exp_– fluorescence in the experimental wells after subtraction of the measured background plasma fluorescence (I_b(m)_); and k–the coefficient of approximation.

For fluorescence in the calibration wells the following equation is correct:


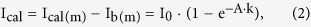


where I_cal(m)_ is the total measured fluorescence in the calibration wells; I_cal_– fluorescence in the calibration wells without a background plasma fluorescence, and A–the AMC concentration in the calibration wells.

The real concentration of AMC in each time point may be calculated from equations (1) and (2) (at given coefficient k):


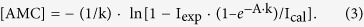


**Figure 8:**
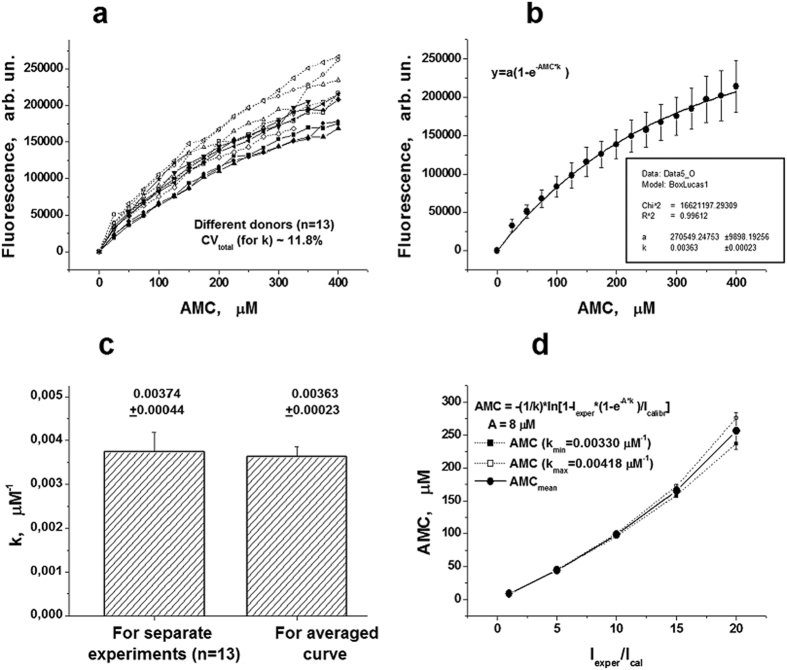
Calculation of averaged coefficient k for correction of an “inner filter” effect. (**a**) Experimental calibration curves observed in different donors’ plasma (n = 13). (**b**) The average calibration curve (mean ± SD, n = 13) and its approximation by function (1). (**c**) Comparison of coefficients k, calculated as mean value of all the obtained individual k (n = 13), or as approximation coefficient for averaged calibration curve. (**d**) Dependence of the errors for calculation of the AMC concentrations on ratio I_exper_/I_calibr_ (for minimal and maximal k values which are equal to 0.00330 and 0.00418 μM^−1^, respectively); the mean value ± SD (n = 2) for these calculated AMC concentrations is also presented. I_exper_ and I_calibr_- the intensities of fluorescence in the experimental and calibration wells, respectively.

The calibration curves measured in 13 plasmas of healthy donors are presented in Fig. 8a. It was shown that the shape of the each curve may be described by the function (1). The averaged calibration curve (mean ± SD, n = 13) and its approximation using this function is presented in Fig. 8b. The mean value of k may be obtained by averaging out all the individual k, calculated for each calibration curve (Fig. 8a), or it may be estimated from average curve (Fig. 8b). These values were similar enough (Fig. 8c), and were equal to 0.00374 ± 0.00044 or to 0.00366 ± 0.00022 μM^−1^, respectively.

Despite the fact that the values of the approximation coefficients (k) in different plasma may vary (CV_total_ for k ~ 11.8%), it does not lead to significant differences in the results calculated using equation (3) in the certain plasma. The relative experimental error (CV_error_) for k determination was calculated in separate experiments with pool of 5 donors’ plasma. The similar calibration curves were measured in 5 independent runs with this plasma pool. The mean CV_error_ was equal to ~8%. The inter-individual coefficient of variation (CV_ii_) for k was calculated according to^35^ and it appeared to be equal to ~8.7%. The calculated AMC concentration, which may be obtained using the minimal and maximal possible values of k (in SD interval) does not differ by more than 5–6% for the highest really observed fluorescence intensity (~90000–100000 arb. un.) (Fig. 8d). This situation corresponds to the value of a ratio I_exp_/I_cal_ equal to ~15. Therefore, then we used the average k value for all the investigated samples.

It should be noted that the coefficients k can be accurately calculated for the each plasma at the proviso that two calibration AMC concentrations are used. The simple equation (4) is correct, if these concentrations differ in magnitude by 2 times.

In this case:


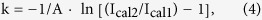


where I_cal1_ and I_cal2_– intensity of fluorescence (without a background plasma fluorescence) in the calibration wells with AMC concentration of A and 2A, respectively.

However in this study the calculation of the “inner filter” effect was conducted using the average value of k (i.e., with an accuracy of 5–6%).

#### Calculation of a total thrombin activity present in plasma

Knowing the rate of accumulation of a product of substrate cleavage in a sample at each time point and considering that the reactions of hydrolysis for this substrate under the action of thrombin and α_2_MT complex follow Michaelis-Menten kinetics, it is possible to calculate the appropriate conditional total activity of thrombin in plasma from the following equation (5):


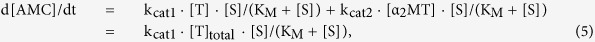


where [T]_total_– the conditional total concentration of thrombin in the sample, which represents the sum of concentrations of the free enzymes and the enzyme-substrate complexes. It may be described by formula (6):


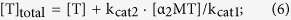


where [T] and [α_2_MT] are the concentrations of the free thrombin +TS complex, and complex of thrombin with α_2_-macroglobulin +α_2_MTS complex, respectively; K_M_, k_cat1_ and k_cat2_ are the kinetic constants of thrombin and α_2_MT complex in relation to the fluorogenic substrate (the given kinetic constants for thrombin were previously determined as follows: K_M_ = 156 μМ, k_cat1_ = 46 min^−1 ^38, and the K_M_ value for α_2_MT was approximately equal to K_M_ for thrombin (160 μM)); [S] is the concentration of the substrate in a sample in the corresponding time point. Since the sum of [TS] and [α_2_MTS] is much lesser, than S_0_, the [S] value may be calculated from the initial substrate concentration (S_0_) and current [AMC] concentration by equation (7):


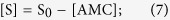


From (5) and (7) it may be finally obtained:


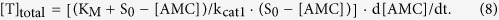


It should be noted that the equation (8) makes it possible to calculate the sum of the concentrations of free enzymes and the enzyme-substrate complexes. The use of this equation allows us to compare the results obtained with our modified method and the CAT, which also calculates this parameter. If it is necessary to calculate the concentrations of the free enzymes, the resulting value [T]_total_ should be multiplied by a coefficient K_M_/(K_M_ + S).

#### Calculation of α_2_MT contribution in the conditional total thrombin activity

This calculation did not depend on a mode of calibration of the fluorescent signal, and was carried out absolutely the same way as it has been described previously in the study^39^. We supposed that binding α_2_-macroglobulin (α_2_MG) with thrombin is irreversible and that the rate of α_2_MT complex accumulation is proportional to the concentration of thrombin in each time point:


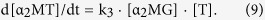


It may be supposed that [α_2_MG] ≈ constant, since [α_2_MT] is much lesser than initial [α_2_MG] concentration.

Turning to the finite differences in the equations (9) and (6) we obtain:


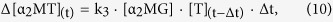


and


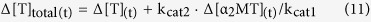


where indexes (t − Δt) and (t) correspond to the time moments (t − Δt) and t, respectively.

Therefore, finally the equation for increment of the sum concentration of free thrombin plus thrombin-substrate complex on each time step may be obtained:


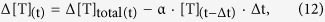


where


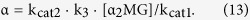


The value of α is selected for each plasma sample individually, based on the condition that at the final part of the thrombin generation curve, when the rate of the product accumulation goes out to the stationary level, the [T] = 0, and the cleavage of the substrate is completely determined only by α_2_MT complex. The special template was constructed in the Origin 6.0 (Microcal Software, USA) for selection of α value using spreadsheet^40^.

## Additional Information

**How to cite this article**: Gribkova, I. V. *et al*. The modification of the thrombin generation test for the clinical assessment of dabigatran etexilate efficiency. *Sci. Rep*. **6**, 29242; doi: 10.1038/srep29242 (2016).

## References

[b1] Eisert W. G. . Dabigatran: an oral novel potent reversible nonpeptide inhibitor of thrombin. Arterioscler Thromb Vasc Biol. 30, 1885–1889 (2010). 20671233 10.1161/ATVBAHA.110.203604

[b2] Baetz B. E. & Spinler S. A. Dabigatran etexilate: an oral direct thrombin inhibitor for prophylaxis and treatment of thromboembolic diseases. Pharmacotherapy 28, 1354–1373 (2008). 18956996 10.1592/phco.28.11.1354

[b3] Stangier J. . Pharmacokinetic profile of the oral direct thrombin inhibitor dabigatran etexilate in healthy volunteers and patients undergoing total hip replacement. J Clin Pharmacol 45, 555–563 (2005). 15831779 10.1177/0091270005274550

[b4] Trocóniz I. F., Tillmann C., Liesenfeld K. H., Schäfer H. G. & Stangier J. Population pharmacokinetic analysis of the new oral thrombin inhibitor dabigatran etexilate (BIBR 1048) in patients undergoing primary elective total hip replacement surgery. J Clin Pharmacol 47, 371–382 (2007). 17322149 10.1177/0091270006297228

[b5] Van Es N. & Buller H. R. Using direct oral anticoagulants (DOACs) in cancer and other high-risk populations. Hematology Am Soc Hematol Educ Program 2015, 125–131 (2015). 26637711 10.1182/asheducation-2015.1.125

[b6] Schulman S. . Treatment with dabigatran or warfarin in patients with venous thromboembolism and cancer. Thromb Haemost 114, 150–157 (2015). 25739680 10.1160/TH14-11-0977

[b7] Wienen W., Stassen J.-M., Priepke H., Ries U. J. & Hauel N. *In-vitro* profile and *ex-vivo* anticoagulant activity of the direct thrombin inhibitor dabigatran and its orally active prodrug, dabigatran etexilate. Thromb Haemost 98, 155–162 (2007). 17598008

[b8] Kyriakou E. . Laboratory assessment of the anticoagulant activity of dabigatran. Clin Appl Thromb Hemost 21, 434–445 (2015). 25525048 10.1177/1076029614564209

[b9] Stangier J., Rathgen K., Stähle H., Gansser D. & Roth W. The pharmacokinetics, pharmacodynamics and tolerability of dabigatran etexilate, a new oral direct thrombin inhibitor, in healthy male subjects. Br J Clin Pharmacol 64, 292–303 (2007). 17506785 10.1111/j.1365-2125.2007.02899.x PMC2000643

[b10] Stangier J. Clinical pharmacokinetics and pharmacodinamics of the oral direct thrombin inhibitor Dabigatran Etexilate. Clin Pharmacokinet 47, 285–295 (2008). 18399711 10.2165/00003088-200847050-00001

[b11] Douxfils J. . Impact of dabigatran on a large panel of routine or specific coagulation assays. Thromb Haemost 107, 985–997 (2012). 22438031 10.1160/TH11-11-0804

[b12] Spannagl M. . Dabigatran therapy–perioperative management and interpretation of coagulation tests. Hamostaseologie 32, 294–305 (2012). 23114798 10.5482/ha-2012030004

[b13] Ten Cate H. New oral anticoagulants: discussion on monitoring and adherence should start now! Thromb J 11, 8 (2013). 23809888 10.1186/1477-9560-11-8 PMC3716685

[b14] Ten Cate H. Monitoring new oral anticoagulants, managing thrombosis, or both? Thromb Haemost 107, 803–805 (2012). 22437154 10.1160/TH12-03-0130

[b15] Hapgood G., Butler J., Malan E., Chunilal S. & Tran H. The effect of dabigatran on the activated partial thromboplastin time and thrombin time as determined by the Hemoclot thrombin inhibitor assay in patient plasma samples. Thromb Haemost 110, 308–315 (2013). 23783268 10.1160/TH13-04-0301

[b16] Montaruli B. . Dabigatran overdose: case report of laboratory coagulation parameters and hemodialysis of an 85-year-old man. Blood Coagul Fibrinolysis 26, 225–229 (2015). 25629417 10.1097/MBC.0000000000000221

[b17] Chin P. K. . Coagulation assays and plasma fibrinogen concentrations in real-world patients with atrial fibrillation treated with dabigatran. Br J Clin Pharmacol 78, 630–638 (2014). 24592919 10.1111/bcp.12366 PMC4243913

[b18] Dietrich K., Stang L., van Ryn J. & Mitchell L. G. Assessing the anticoagulant effect of dabigatran in children: An *in vitro* study. Thromb Res 135, 630–635 (2015). 25715905 10.1016/j.thromres.2015.01.017

[b19] Dager W. E., Gosselin R. C., Kitchen S. & Dwyre D. Dabigatran effects on the international normalized ratio, activated partial thromboplastin time, thrombin time, and fibrinogen: a multicenter, *in vitro* study. Ann Pharmacother 46, 1627–1636 (2012). 23232017 10.1345/aph.1R179

[b20] Lippi G. & Favoloro E. J. Recent guidelines and recommendations for laboratory assessment of the direct oral anticoagulants (DOACs): is there consensus? Clin Chem Lab Med 53, 185–197 (2015). 25241734 10.1515/cclm-2014-0767

[b21] Van Ryn J., Grottke O. & Spronk H. Measurement of dabigatran in standardly used clinical assays, whole blood viscoelastic coagulation, and thrombin generation assays. Clin Lab 34, 479–501 (2014). 10.1016/j.cll.2014.06.008 25168938

[b22] Chin P., Wright D., Patterson D., Doogue M. & Begg E. A proposal for dose-adjustment of dabigatran etexilate in atrial fibrillation guided by thrombin time. Br J Clin Pharmacol 78, 599–609 (2014). 24592851 10.1111/bcp.12364 PMC4243910

[b23] Van Ryn J. . Dabigatran etexilate–a novel, reversible, oral direct thrombin inhibitor: interpretation of coagulation assays and reversal of anticoagulant activity. Thromb Haemost 103, 1116–1127 (2010). 20352166 10.1160/TH09-11-0758

[b24] Dinkelaar J., Patiwael S., Harenberg J., Leyte A. & Brinkman H. J. M. Global coagulation tests: their applicability for measuring direct factor Xa- and thrombin inhibition and reversal of anticoagulation by prothrombin complex concentrate. Clin Chem Lab Med 52, 1615–1623 (2014). 24902009 10.1515/cclm-2014-0307

[b25] Hemker H. C. . The calibrated automated thrombogram (CAT): a universal routine test for hyper- and hypocoagulability. Pathophysiol. Haemost. Thromb 32, 249–253 (2002). 10.1159/000073575 13679651

[b26] Sharrock N. E. . Changes in circulatory indices of thrombosis and fibrinolysis during total knee arthroplasty performed under tourniquet. J Arthroplasty 10, 523–528 (1995). 8523013 10.1016/s0883-5403(05)80155-x

[b27] Al Dieri R., Alban S., Beguin S. & Hemker H. C. Thrombin generation for the control of heparin treatment, comparison with the activated partial thromboplastin time. J Thromb Haemost 2, 1395–1401 (2004). 15304046 10.1111/j.1538-7836.2004.00798.x

[b28] Al Dieri R., Alban S., Beguin S. & Hemker H. C. Fixed dosage of low-molecular-weight heparins causes large individual variation in coagulability, only partly correlated to body weight. J Thromb Haemost 4, 83–89 (2006). 16409456 10.1111/j.1538-7836.2005.01672.x

[b29] Gatt A. . Thrombin generation assays are superior to traditional tests in assessing anticoagulation reversal *in vitro*. Thromb Haemost 100, 350–355 (2008). 18690358

[b30] Green L. . The effect of total hip/knee replacement surgery and prophylactic dabigatran on thrombin generation and coagulation parameters. Thromb Res 130, 775–779 (2012). 22245224 10.1016/j.thromres.2011.12.023

[b31] Helin T. A. . From laboratory to clinical practice: Dabigatran effects on thrombin generation and coagulation in patient samples. Thromb Res 136, 154–160 (2015). 25981140 10.1016/j.thromres.2015.04.032

[b32] Wagenvoord R. J., Deinum J., Elg M. & Hemker H. C. The paradoxical stimulation by a reversible thrombin inhibitor of thrombin generation in plasma measured with thrombinography is caused by alpha-macroglobulin-thrombin. J Thromb Haemost 8, 1281–1289 (2010). 20180821 10.1111/j.1538-7836.2010.03822.x

[b33] Sinauridze E. I. . New Synthetic Thrombin Inhibitors: Molecular Design and Experimental Verification. PLoS ONE 6, e19969 (2011). 21603576 10.1371/journal.pone.0019969 PMC3095642

[b34] Wong P. C., White A. & Luettgen J. Inhibitory effect of apixaban compared with rivaroxaban and dabigatran on thrombin generation assay. Hospital Practice 41, 19–25 (2013). 23466964 10.3810/hp.2013.02.1009

[b35] Bloemen S., Hemker H. C. & Al Dieri R. Large inter-individual variation of the pharmacodynamic effect of anticoagulant drugs on thrombin generation. Haematologica 98, 549–554 (2013). 23100275 10.3324/haematol.2012.073601 PMC5490471

[b36] Hemker H. C. . Calibrated automated thrombin generationmeasurement in clotting plasma. Pathophysiol Haemost Thromb 33, 4–15 (2003). 12853707 10.1159/000071636

[b37] Rogan L on behalf of Lancashire and Cumbria Health Economy New Medicines and Treatments Group. Guidance for prescribing of Dabigatran (Pradaxa) Rivaroxaban (Xarelto) and Apixaban (Eliquis) in Patients with Non-Valvular, A. F., 2012. Available at: http://www.cumbria.nhs.uk/ProfessionalZone/MedicinesManagement/Guidelines/Prescribing-Guidance-for-NOACs.pdf (Accessed April 28, 2016).

[b38] Tarandovskiy I. D., Artemenko E. O., Panteleev M. A., Sinauridze E. I. & Ataullakhanov F. I. Antiplatelet agents can promote two-peaked thrombin generation in platelet rich plasma: mechanism and possible applications. PLoS ONE 8, e55688 (2013). 23405196 10.1371/journal.pone.0055688 PMC3566002

[b39] Hemker H. C. & Kremers R. Data management in thrombin generation. Thromb. Res. 131, 3–11 (2013). 23158401 10.1016/j.thromres.2012.10.011

[b40] Sinauridze E. I. . Platelet microparticle membranes have 50- to 100-fold higher specific procoagulant activity than activated platelets. Thromb. Haemost 97, 425–434 (2007). 17334510

